# Comparison of flexible fixation and screw fixation for isolated Lisfranc ligament injuries: A protocol for a meta-analysis of comparative studies

**DOI:** 10.1097/MD.0000000000031233

**Published:** 2022-10-21

**Authors:** Wenxuan Guo, Wenhuan Chen, Jinsheng Yu, Fan Wu, Wenqiang Qian, Siyuan Zhuang, Kun Tian, Rujie Zhuang, Yu Pan

**Affiliations:** a Department of Orthopaedics, The First Affiliated Hospital of Zhejiang Chinese Medical University, Hangzhou, Zhejiang, China; b The First Clinical College, Zhejiang Chinese Medical University, Hangzhou, Zhejiang, China; c Third Clinical Medical School, Guangzhou University of Chinese Medicine, District Baiyun, Guangzhou, Guangdong, China.

**Keywords:** flexible fixation, Lisfranc injury, protocol, screw fixation

## Abstract

The frequency of isolated Lisfranc ligament (ILL) injuries has been increasing recently with the increase in low-energy trauma resulting from sports injuries. For ILL injuries, the optimal method of fixation still remains controversial. The traditional fixation method is achieved by trans-articular screws, but recently, dorsal bridge plates and suture button (SB) fixation have become alternatives. Some biomechanical studies have showed that SB fixation can provide adequate strength compared to trans-articular screws. Therefore, a meta-analysis is imperative to provide evidence on whether flexible fixation is comparable to screw fixation for treatment of ILL injuries.

**Methods:**

We will conduct a comprehensive literature search in PubMed, Cochrane Library, EMBASE and Web of Science databases and for comparative studies. We will apply the risk-of-bias tool of the Cochrane Collaboration for Randomized Controlled Trials to assess the methodological quality. Risk-of-Bias Assessment Tool for Non-randomized Studies was used to evaluate the quality of comparative studies. Statistical analysis will be conducted using RevMan 5.4 software (Cochrane Collaboration, London, England).

**Results:**

This systematic review will evaluate the functional outcomes and radiographic results of flexible fixation for treatment of ILL injuries.

**Conclusion:**

The conclusion of this study will provide evidence for judging whether flexible fixation is superior to screw fixation for treatment of ILL injuries.

## 1. Introduction

The incidence of Lisfranc injuries is 14/100,000 person-years, with high-energy injury accounting for 31%.^[1]^ However, about 20% to 40% of the injuries were misdiagnosed initially on primary radiographs.^[2,3]^ Untreated Lisfranc injuries can lead to chronic foot disability and deformity.^[4]^ With the increasing awareness of these injuries and the popularity of magnetic resonance imaging, computed tomography scans, weight bearing radiographs and stress fluoroscopy, the incidence rate of Lisfranc injury is getting higher.^[1,5–8]^ Meanwhile, the frequency of isolated Lisfranc ligament (ILL) injuries has been increasing recently with the increase in low-energy trauma resulting from sports injuries.^[9]^

For ILL injuries, the optimal method of fixation still remains controversial.^[10]^ The traditional fixation method is achieved by trans-articular screws, but recently, dorsal bridge plates and suture button (SB) fixation have become alternatives.^[10–12]^Some biomechanical studies have showed that SB fixation can provide adequate strength compared to trans-articular screws.^[13,14]^ The rationale for flexible Fixation is to obtain more physiologic movement of the joint during loading while maintain the required reduction.^[15]^ At the same time, flexible Fixation avoids the potential morbidity of fractured screws and the need to remove hardware, and reduces iatrogenic damage to articular cartilage.^[16,17]^

However, lack of high-level guidelines and evidence-based research have affected decision-making and clinical application of fixation in ILL injuries. Therefore, a meta-analysis is imperative to provide evidence on whether flexible fixation is comparable to screw fixation for treatment of ILL injuries, owing to an increase in related studies that have been published in recent years. In this study, we seek to conduct a meta-analysis of relevant studies to evaluate and compare functional outcomes and complication rates between flexible fixation and screw fixation for treatment of ILL injuries. Our findings are expected to provide a reference to guide future treatment options.

## 2. Methods

### 2.1. Study registration

We have prospectively registered this research at the international prospective register of systematic reviews (PROSPERO)-Registration number: CRD42022353815. We performed this protocol based on the Preferred Reporting Items for Systematic Review and Meta-analysis Protocols (PRISMA-P) statement guidelines.^[18]^

### 2.2. Inclusion criteria

#### 2.2.1. Type of participants.

The participants diagnosed as closed Lisfranc injuries will be included regardless their country, ethnicity, sex, occupation and mechanism of injury.

#### 2.2.2. Type of interventions.

In the experimental group, all patients received flexible Fixation (such as SB) to fix Lisfranc ligament. In the control group, all patients received static fixation (such as trans-articular screws fixation) to fix Lisfranc ligament.

#### 2.2.3. Type of outcome measurements.

##### 2.2.3.1. Primary outcomes.

American Orthopaedic Foot and Ankle Society midfoot score^[19]^ and Diastasis between the first and second metatarsals will be defined as the primary outcomes to assess the function and fixation stability.

##### 2.2.3.2. Secondary outcomes.

Visual analog scale, Plantar Foot Pressure, complications will be defined as secondary outcomes.

#### 2.2.4. Type of studies.

We will include comparative studies which published in Chinese or English, such as randomized controlled trials, retrospective studies and cohort studies. Review, case reports, experimental studies, expert experience, animal studies and conference abstracts will be excluded.

### 2.3. Search strategy

We will search the PubMed, Cochrane Library, EMBASE and Web of Science databases from the inception dates to August 1, 2021, using the keywords “Midfoot”, “Lisfranc”, “Fixation”, “Screw”, “Suture Button” and “Tightrope”. The search strategy in PubMed is shown in Table 1. In addition, the reference lists of previously published systematic reviews of fixation of Lisfranc injuries were manually examined for further pertinent studies.

**Table 1:** Search strategy of PubMed.

Number	Search terms
#1	Midfoot
#2	Lisfranc
#3	Fixation
#4	Screw
#5	Suture button
#6	Tightrope
#7	#1 or #2
#8	#3 or #4 or #5 or #6
#9	#7 or #8

### 2.4. Study selection

Two independent researchers (W-XG, W-HC) screened the study titles and abstracts according to the inclusion criteria. The full text of the studies potentially meeting the eligibility criteria were retrieved for a more detailed read to make a final decision regarding inclusion.

### 2.5. Data extraction and management

**Figure 1.**
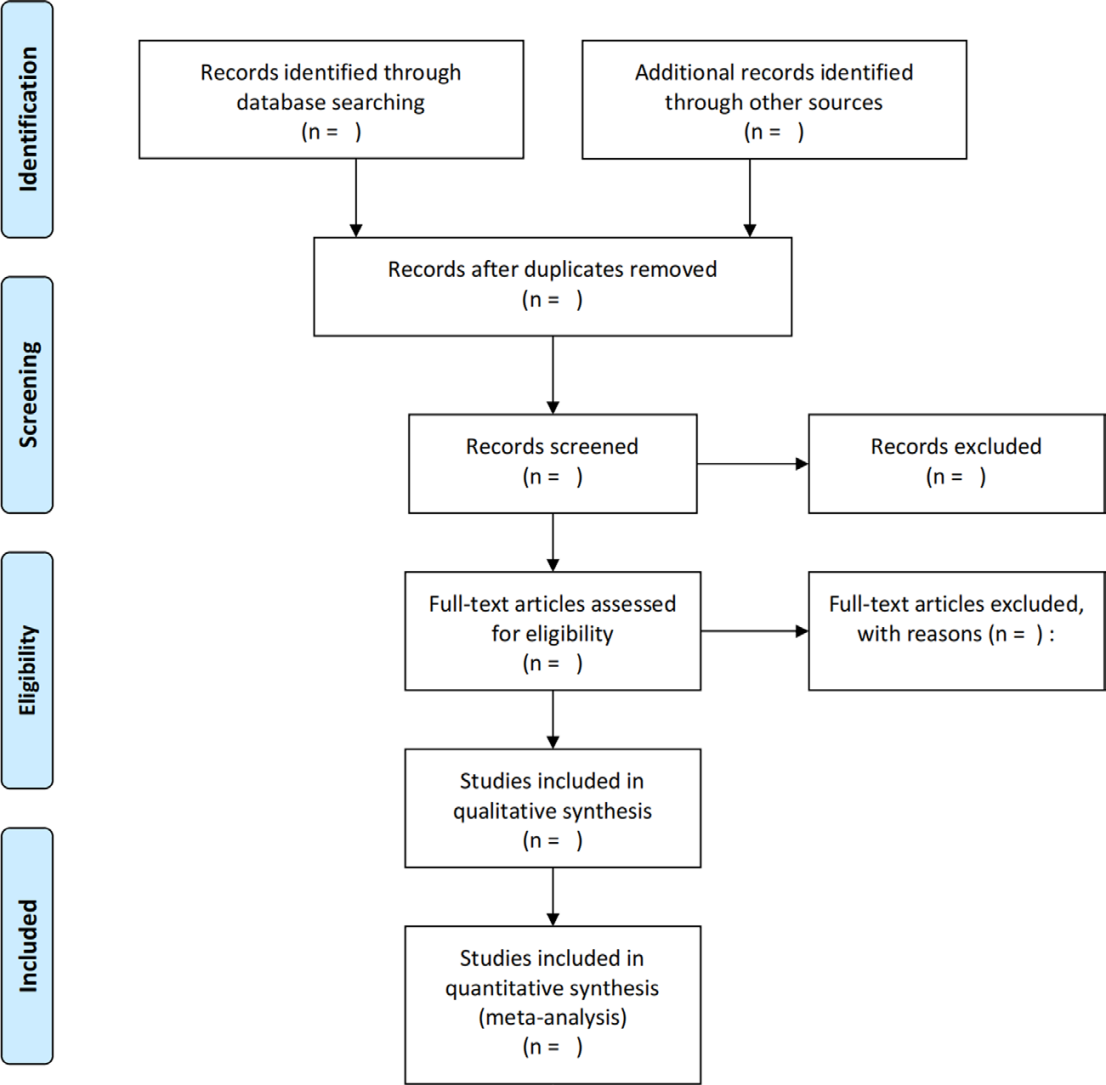
PRISMA flow diagram. PRISMA-P = preferred reporting items for systematic review and meta-analysis protocols.

The following data were extracted: lead author; publication year; country of origin; study design; sample size; age; injury type; fixation technique; outcome measures and complications. Any differences of opinion will be resolved through group discussion or consultation with a third reviewer. When relevant data is not reported, we will contact the author via email or other means to obtain missing data. The Preferred Report items for the System Review and Meta-analysis (PRISMA) flow diagram (Fig. 1) will be filled out after the screening study is completed to provide specific information.

### 2.6. Risk of bias assessment

Two independent (W-XG, W-HC) investigators evaluated the quality of the included studies. The Cochrane Collaboration Risk of Bias Tool^[20]^ was used to evaluate the quality of the randomized controlled trials. The methodological quality of the non-randomized studies was assessed using the Risk-of-Bias Assessment Tool for Non-randomized Studies.^[21]^ The level of evidence was assessed according to the Oxford Centre for Evidence-based Medicine Levels of Evidence.

### 2.7. Data synthesis

Statistical analysis will be conducted using RevMan 5.4 software (Cochrane Collaboration). The mean difference will be used as the effect analysis statistic for continuous variables, while the risk ratio will be used as the effect analysis statistic for categorical variables. We will also calculate 95% confidence interval for each statistic, and summarize statistical heterogeneity among summary data using the *I*^2^ statistic. Cases with *I*^2^ ≤ 50% will not be considered to have significant heterogeneity, thus a fixed-effects model will be applied for meta-analysis. In cases where there is statistical heterogeneity among studies, we will further analyze the source of heterogeneity. A random-effects model will be used to pool the data, after excluding the obvious source of clinical heterogeneity, and in cases where obvious clinical heterogeneity exists, the researchers will perform subgroup, sensitivity or only descriptive analyses. Study-specific and pooled estimates will be graphically presented using forest plots, and *P* < .05 considered statistically significant.

### 2.8. Subgroup analysis

Subgroup analysis according to the age, injury type and the classifications for lisfranc injury will be performed to find the source of heterogeneity when significant clinical heterogeneity is observed.

### 2.9. Sensitivity analysis

Sources of heterogeneity were assessed by sensitivity analysis, by excluding studies of low quality or small sample size, if the heterogeneity did not change significantly, the results were robust. Otherwise, the excluded studies may have been source of heterogeneity.

### 2.10. Publication bias

In this study, fewer than 10 included studies were evaluated for publication bias using funnel plot, otherwise Egger regression test would be used.^[22,23]^

### 2.11. Ethics and dissemination

No ethical approval is required because the study will be a review of literature and will not obtain data from a single patient. We will publish our findings through a peer-reviewed journal.

## 3. Discussion

The purpose of this meta-analysis aims to assess the functional outcomes, radiographic outcomes and complications between flexible Fixation and screw fixation for treatment of ILL injuries. There is growing interest in the use of flexible fixation devices to treat ligamentous Lisfranc injuries.^[16]^ To our best knowledge, this study is the first systematic review and meta-analysis on this topic, integrating the latest and most comprehensive clinical evidence in this field, hoping to provide helpful evidence for the patients, clinician and inspire more peer experts and doctors to carry out relevant research as much as possible in the future.

## Author contributions

**Conceptualization:** Wenxuan Guo, Kun Tian.

**Formal analysis:** Wenhuan Chen.

**Investigation:** Wenhuan Chen, Siyuan Zhang.

**Methodology:** Fan Wu, Siyuan Zhang.

**Project administration:** Fan Wu.

**Resources:** Fan Wu.

**Supervision:** Wenqiang Qian.

**Software:** Fan Wu.

**Validation:** Wenqiang Qian.

**Visualization:** Wenqiang Qian.

**Writing – original draft:** Wenxuan Guo.

**Writing – review & editing:** Yu Pan, Rujie Zhuang.
